# Lipid metabolism in the resuscitation of dormant mycobacterium tuberculosis: molecular resilience, host hijacking, and clinical opportunities

**DOI:** 10.3389/fcimb.2026.1838865

**Published:** 2026-08-05

**Authors:** Chunze Wang, Pei Liu, Jingyang Tang, Lei Xu, Zhilong Liu

**Affiliations:** Department of Laboratory Medicine, Dazhou Integrated Traditional Chinese Medicine & Western Medicine Hospital, Dazhou Second People’s Hospital, Dazhou, China

**Keywords:** host-pathogen interaction, ICE-TB continuous spectrum, lipid metabolism, mycobacterium tuberculosis, reactivation and latency

## Abstract

Latent infection and reactivation of Mycobacterium tuberculosis (*Mtb*) represent key obstacles to the global strategy to end tuberculosis. The 2024 ICE-TB international consensus redefined tuberculosis as a continuum of pathological states that includes subclinical forms, with lipid metabolism being the core mechanism driving *Mtb*’s transition across different disease stages. This review systematically elucidates the molecular mechanisms by which *Mtb* maintains survival through lipidome remodelling under restrictive microenvironments (phosphate deprivation, oxidative stress), with a focus on the synergistic roles of key regulatory factors such as MtrA, DosR and OmamC in sensing environmental signals and initiating the reactivation process. At the host-pathogen interaction level, this paper discusses *Mtb*’s pathogenic strategies, including the construction of a lipid-enriched microenvironment by hijacking lysosomal function through the secretion of 1-TbAd, and the induction of systemic insulin resistance via the PPE2 protein. Furthermore, this review evaluates the clinical utility of biomarkers based on lipid metabolic profiles in predicting the risk of relapse, providing a theoretical basis for the development of novel intervention strategies targeting lipid metabolic pathways.

## Introduction

Tuberculosis (TB), caused by the *Mtb* complex, remains one of the deadliest infectious diseases worldwide. According to the World Health Organization (WHO) Global Tuberculosis Report 2025, approximately 10.7 million people fell ill with TB, and 1.23 million died in 2024 (World Health Organization, 2025). However, this heavy disease burden is not solely driven by new infections; a significant proportion of active cases arise from the reactivation of latent tuberculosis infection (LTBI). The LTBI reservoir, affecting approximately one-quarter of the global population, constitutes a massive pool contributing to the persistent global burden of disease, with active cases ultimately driving sustained transmission (Houben and Dodd, 2016; World Health Organization, 2025). This condition is not a static state of absolute bacterial dormancy but rather a dynamic equilibrium involving immune surveillance and metabolic competition (Boom et al., 2021; Verma et al., 2022). Understanding this dynamic process relies on a redefined perception of the TB disease spectrum,which shifts away from a strict binary model to a continuous spectrum where traditional ‘latent’ TB is often conceptually absent (Behr et al., 2018; Migliori et al., 2021).

**Figure 1:**
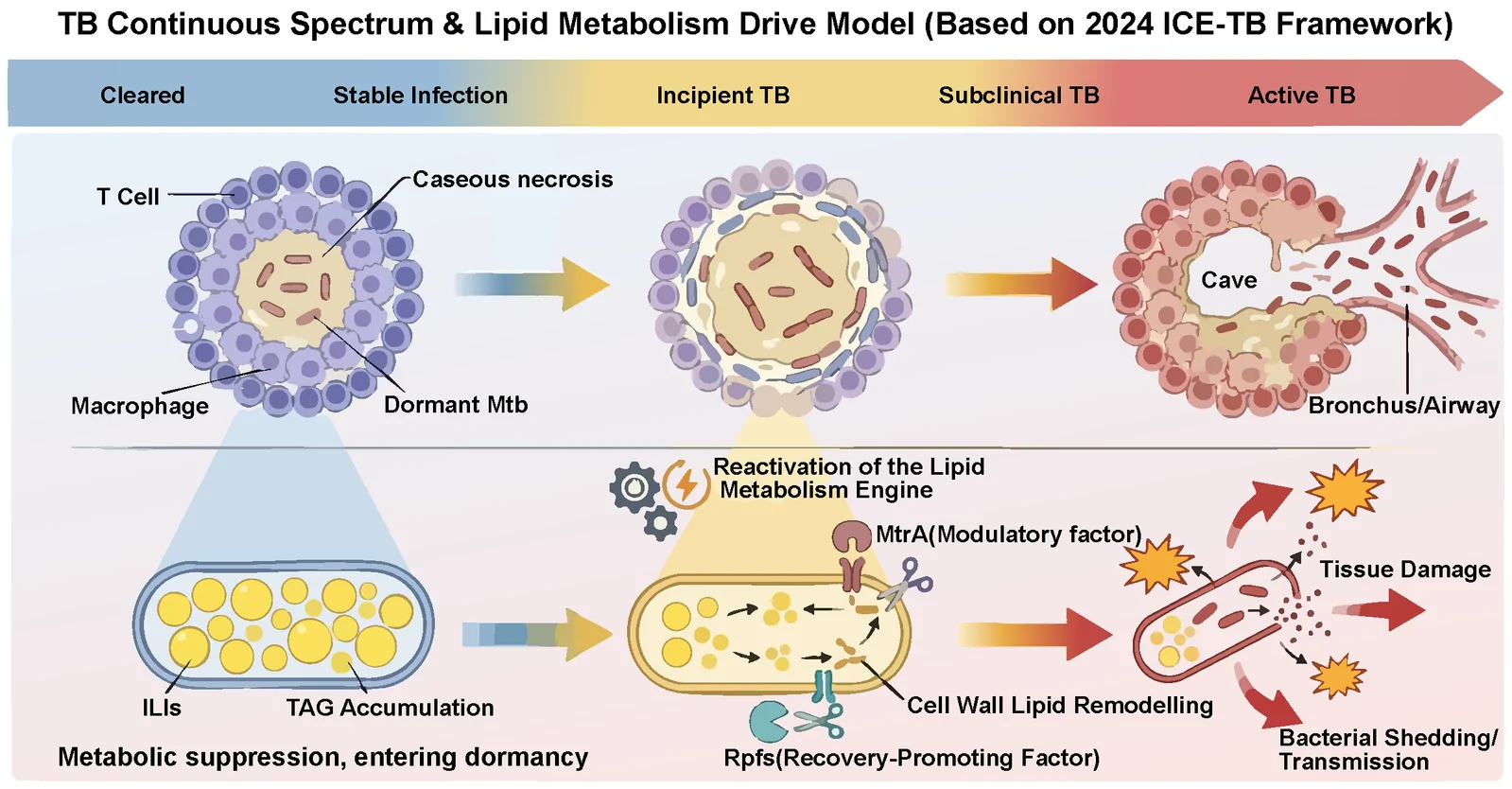
Continuum of tuberculosis and lipid metabolism-driven model. This image illustrates the progression of the disease within the ICE-TB framework in 2024. In the “stable infection state”, Mtb enters dormancy by accumulating triacylglycerols (TAGs) to form lipid inclusions (ILIs); as the disease transitions to the ‘initiation state’ and ‘subclinical state’, the lipid metabolism engine is reactivated, with MtrA and Rpfs driving cell wall lipid remodelling, ultimately leading to tissue damage and bacterial shedding.

In recent years, the research paradigm has undergone a significant transformation. The “International Consensus for Early TB (ICE-TB)” published in 2024 challenged the binary “latent vs. active” framework that persisted for the past half-century, supporting instead a new classification system based on a continuous disease spectrum (Denholm et al., 2024). This consensus categorizes disease evolution into five stages: cleared infection, stable infection, incipient TB, subclinical TB, and clinical TB (**Figure 1**). Within this continuous spectrum, reactivation represents the transition from low-metabolic homeostasis under immune suppression to pathological expansion. Notably, subclinical TB, despite the absence of obvious symptoms, contributes to over 50% of community transmission and represents a critical blind spot in current control strategies (Frascella et al., 2021; Emery et al., 2023). However, the reclassification of the disease spectrum only provides a “roadmap”; the molecular engine driving these transitions remains a core question to be answered. Increasing evidence points toward a common metabolic node: lipid metabolism.

Lipid metabolic regulation is not only a survival strategy for *Mtb* to cope with host microenvironmental restrictions but also a core mechanism driving stage transitions within the disease spectrum (Devlin et al., 2024; Lin et al., 2024; Padilla-Gomez et al., 2025). *Mtb* is a highly evolved “lipid expert”, with its cell envelope comprising nearly 40% of its dry weight in complex lipids. These lipids not only form a physical barrier against host immune attacks but also sense microenvironmental stresses (such as phosphate limitation and oxidative stress) by remodeling cell membrane components, thereby triggering resuscitation-related signaling pathways (Gray et al., 2025). LINC02528 has been identified as a central checkpoint through which Mtb manipulates host immune metabolism (Xu et al., 2025). This discovery not only validates *Mtb*’s active defence strategies at the molecular level but also provides a key target for the development of host-directed therapies (HDTs) targeting the lncRNA-mitochondrial axis (Xu et al., 2025). Therefore, systematically elucidating the lipid dynamics of *Mtb* during latency and reactivation is of great significance for uncovering its pathogenic essence and developing novel diagnostic and therapeutic strategies (Menon et al., 2024; Sturm et al., 2024; Sun et al., 2025). This review integrates recent interdisciplinary research progress to systematically explore the driving role of host and pathogen lipid metabolism in TB reactivation and its potential interventional value at the molecular, cellular, and systemic levels.

## Metabolic reprogramming and survival resilience in extreme restrictive niches

Upon invading macrophage phagosomes or granuloma cores, *Mtb* must implement precise physiological regulation within a harsh restrictive environment. This involves not only extreme conservation of essential elements but also proactive countering of host immune pressure.

### Phosphate homeostasis and lipidome remodeling

Active response to essential element scarcity is *Mtb*’s primary defense for maintaining physiological functions. Inorganic phosphate (*P_i_*), a core element for nucleic acid synthesis and energy metabolism, often drops below 10*μM* in host phagosomes—far below the threshold required for normal bacterial growth. Using high-resolution mass spectrometry, previous studies performed the first deep analysis of the *Mtb* lipidome, revealing a RegX3-driven “dephosphorylation” remodeling mechanism under *P_i_* starvation involving over 1500 lipid species (Gray et al., 2025) (**Table 1**). The core strategy of this remodeling lies in “phosphorus-free” lipid substitution: *Mtb* systematically replaces traditional phospholipids with ornithine lipids (OLs) and glucuronosyldiacylglycerols (GlcA-DAGs), maintaining cell membrane physical integrity and surface charge balance even under extreme nutritional deprivation.

**Table 1:** Compensatory lipidome characteristics of Mtb in phosphate-restricted microenvironments.

Alternative lipid class	Chemical structure features	Fold enrichment (*P_i_* restricted vs. replete)	Key physiological functions
Ornithine lipids (OLs)	β-hydroxy fatty acid amide-linked to ornithine	1,020-fold	Replaces phosphatidylethanolamine; maintains membrane charge balance
Glucuronosyldiacylglycerols (GlcA-DAGs)	Glucuronic acid head group linked to DAG	4,100-fold	Core anionic lipid substitute; maintains membrane integrity
O-methyl GlcA-DAGs	Sugar head group with specific O-methylation	120-fold	Regulates membrane hydrophobicity and acid resistance; first discovered in 2025
Trehalose monomycolates (TMMs)	Trehalose linked to long-chain mycolic acids	Significantly detected	Strengthens outer membrane barrier; enhances phenotypic drug resistance

This compensatory synthesis strategy reflects *Mtb*’s optimization capability for limited resources. Simultaneously, *Mtb* actively “plunders” host resources by secreting glycerophosphodiesterase GlpQ1 to degrade and recycle the polar head groups of phospholipids from host pulmonary surfactants, converting them into alternative phosphate sources (Gray et al., 2025). This active acquisition mechanism works synergistically with the membrane lipid substitution strategy, forming the metabolic foundation for *Mtb*’s early lung colonization and long-term latency (Warner et al., 2025). Notably, this lipid remodeling is not an isolated stress response but is deeply coupled with the transition of *Mtb*’s overall metabolic state. Membrane lipid remodeling may be an active regulatory node for *Mtb*’s transition toward low-metabolic latency rather than just a passive adaptation to *P_i_* restriction.

### Redox sensing and the “reductive sink” defense mechanism

Host-derived reactive oxygen species (ROS) and reactive nitrogen species (RNS) are not only major oxidative stresses faced by *Mtb* but also key environmental signals inducing its entry into dormancy. To address this challenge, *Mtb* has evolved a sophisticated redox-sensing system centered on WhiB3. WhiB3 monitors *O_2_* and *NO* concentration fluctuations in the microenvironment via its iron-sulfur cluster and functions as a “metabolic shunt”: upon sensing oxidative stress, WhiB3 directs excess intracellular metabolic reducing equivalents (primarily NADPH) into fatty acid synthesis pathways (Singh et al., 2009; Mehta and Singh, 2019; Barrientos et al., 2022).

This metabolic redirection is termed the “Reductive Sink” mechanism. Its essence lies in converting excess reducing power into triacylglycerol (TAG), which is accumulated and stored within intracellular lipid inclusions (ILIs) (Singh et al., 2009). This mechanism possesses dual survival value: first, by consuming excess reducing equivalents, it effectively blocks free radical-mediated oxidative cascades, protecting bacterial DNA and critical proteins from damage; second, it stores metabolic energy as high-density, chemically stable solid lipids, providing sufficient endogenous fuel reserves for rapid resuscitation once immune pressure subsides (Zheng et al., 2024). This “Reductive Sink” highlights *Mtb*’s proactive metabolic strategy to transform environmental stress into a survival advantage. The formation of ILIs marks the bacterial transition from active proliferation to low-metabolic dormancy, providing the material basis for long-term latency and eventual reactivation.

## Metabolic breakout from quiescence: coupling mtra networks and lipid mobilization

The transition from a non-replicating persistent (NRP) state to a highly active revived state is, in essence, an “all-or-nothing” decision-making process driven by the synergistic action of essential regulatory factors and transport systems. This process requires precise spatiotemporal coordination of genome replication, cell wall remodelling and energy supply; any imbalance at any stage may result in failed revival or metabolic collapse.

### MtrA regulon: commander of genomic replication and envelope reconstruction

At the apex of the resuscitation signal cascade, MtrA (Mycobacterial transcriptional regulator A) occupies a critical master regulatory position. As the sole essential response regulator in *Mtb*’s signal transduction systems, MtrA exhibits remarkable regulatory breadth, with direct targets covering approximately 32.6% of the essential genes across the genome (Gorla et al., 2018).

MtrA constructs a multi-layered regulatory network spanning the cell cycle. At the transcriptional level, it initiates the chromosomal replication program by directly binding the *dnaA* promoter region. At the structural level, it synchronously regulates the peptidoglycan hydrolase *ripA* and the mycolyltransferase *fbpB* (Antigen 85B), coordinating local cell wall softening with dynamic lipid envelope reconstruction (Peterson et al., 2023). This “pleiotropic synergy” ensures a dynamic balance between genomic integrity and cell envelope structural integrity as *Mtb* enters rapid growth. Experimental evidence indicates that abnormal fluctuations in MtrA expression levels lead to impaired metabolic integrity and significantly increase susceptibility to first-line anti-TB drugs, making it a “safety valve” for survival on the eve of resuscitation (Gorla et al., 2018).

### OmamC-Mce1 axis: precision mobilization of lipid “storage and retrieval”

While MtrA restarts the transcriptional program, the lipid mobilization axis provides sustained energy support. Research filled a critical gap in *Mtb*’s lipid uptake mechanism, elucidating the core role of the orphaned membrane protein OmamC in lipid metabolic homeostasis (Chandra et al., 2022).

**Figure 2:**
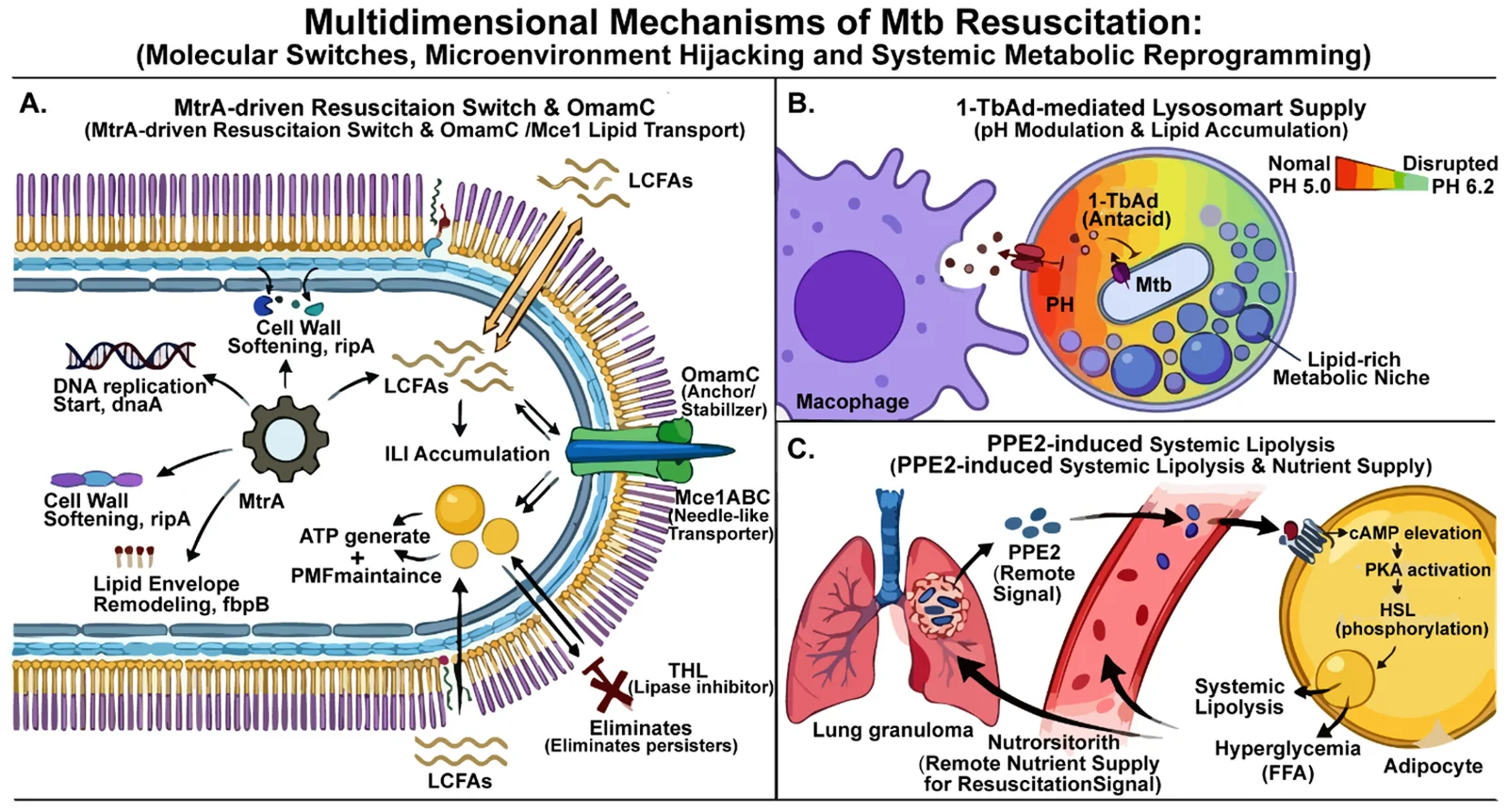
Schematic diagram of the molecular switch for Mtb reactivation and the mechanism of host hijacking. **(A)** Inside the bacterium: MtrA co-regulates DNA replication and lipid envelope synthesis; OmamC transports fatty acids via the Mce1 needle-like complex. **(B)** Host interaction: 1-TbAd acts as an ‘anti-acid agent’ to raise lysosomal pH (5.0 to 6.2), inducing lipid accumulation to form a “metabolic niche”. **(C)** Systemic effects: The PPE2 protein activates the cAMP-PKA-HSL axis, inducing lipolysis and hyperglycaemia, thereby providing remote material supply for reactivation.

OmamC stabilizes and anchors the transmembrane Mce1ABC transporter complex, establishing a lipid transport channel across the entire cell envelope to assist the bacteria in capturing trace long-chain fatty acids (LCFAs) from the environment during carbon starvation. This system exhibits precise bidirectional regulation across different metabolic states: during the nutrient-rich growth phase, OmamC promotes efficient lipid uptake and ILI accumulation; during early resuscitation, it guides the rapid decomposition of TAG within ILIs to provide the minimum electron flow required to maintain the proton motive force (PMF) (Chandra et al., 2022). As metabolic levels rise, OmamC anchors the Mce1ABC needle-like complex to switch the resource acquisition mode to high-efficiency capture of host LCFAs. This precise “storage and retrieval” ensures energy supply-demand matching during state transitions(**Figure 2**). Notably, this mobilization system provides an ideal target for drug intervention. Experiments have shown that inhibiting OmamC-dependent lipid utilization with lipase inhibitors (such as tetrahydrolipstatin, THL) specifically eradicates non-replicating persister populations, opening new directions for treating LTBI and blocking reactivation.

## Host-pathogen interaction: 1-TbAd and the lysosomal “metabolic niche”

*Mtb*’s reactivation success depends heavily on its proactive remodeling of the host cell microenvironment during latency. While foamy macrophages (FM) were traditionally viewed as passive formations, recent research shows that *Mtb* actively secretes virulence factors to hijack host lysosomes, transforming them into “lipid gas stations” (Chandra et al., 2022). The key effector molecule in this process is 1-TbAd, a secreted adenosine derivative that implements “chemical castration” of host lysosomes. 1-TbAd is a weakly basic compound with strong acid affinity that accumulates in acidic lysosomes as an “antacid,” raising the vacuolar *pH* from approximately 5.0 to 6.2 (Buter et al., 2019). This slight *pH* shift leads to the pleiotropic inactivation of approximately 60 acid-dependent hydrolases (such as acid lipases and proteases), accurately mimicking the pathological features of lysosomal storage diseases like Gaucher’s and Wolman’s diseases at the molecular level (Bedard et al., 2023). Impaired lysosomes fail to process ingested LDL and cellular debris, resulting in the abnormal accumulation of cholesteryl esters (CEs) and triacylglycerols (TAGs) within the lysosomal lumen (Aguilar-Ayala et al., 2017). Notably, *Mtb* colonization in host macrophages exhibits a non-random spatial distribution, specifically localizing around host lipid droplets (LDs) via active chemotaxis, forming a “Metabolic Niche” centered on lipid metabolic reprogramming (Silwal et al., 2021; Mukherjee et al., 2022; Bi et al., 2025). This deep hijacking of host lipid pathways is strategically vital. By maintaining spatial proximity to LDs, quiescent bacteria ensure rapid access to these sequestered lipid resources once immune pressure subsides, enabling quick metabolic restart and resuscitation expansion.

## Systemic hijacking: PPE2 protein-induced systemic metabolic disorder

*Mtb*’s ability to regulate host lipid metabolism extends beyond the local infection site to the systemic endocrine system. Recent studies providing molecular explanations for the clinical comorbidity between TB and Type 2 Diabetes (T2DM) have highlighted this (Kumar et al., 2019; Wang et al., 2023; Du et al., 2025; Bisht et al., 2026). A study identified the secreted protein PPE2 as a key molecular driver inducing systemic insulin resistance (Bisht et al., 2026). PPE2 enters distant adipose tissue via the circulatory system (and in some cases crosses the blood-brain barrier), inducing severe lipoatrophy and adipocyte hypertrophy. Its molecular mechanism involves inhibiting phosphodiesterase PDE3B, thereby activating the cAMP-PKA-HSL signal cascade in host adipose tissue. Overactivation of this axis triggers massive lipolysis, elevating circulating non-esterified fatty acids (NEFA) and inducing systemic insulin resistance and hyperglycemia.

This *Mtb*-induced systemic metabolic reprogramming provides a dual advantage for its survival and spread: first, the hyperglycemic microenvironment directly impairs the phagocytic and bactericidal functions of neutrophils and macrophages; second, elevated circulating lipids provide a continuous remote nutrient supply to infection sites throughout the body, significantly increasing the probability of reactivation in extrapulmonary organs. This discovery expands the research paradigm of TB from a local infection to systemic intervention in host metabolic homeostasis, providing a molecular basis for the bidirectional causality of TB-T2DM comorbidity.

## Clinical translation: precision early warning via lipid metabolic fingerprints

The specificity of lipid metabolic alterations provides high-resolution “metabolic fingerprints” for precision TB warning (Lyu et al., 2024; Comella-Del-Barrio et al., 2025; Ding et al., 2025; Sun et al., 2025). Lipidomic analysis is progressively addressing key challenges in traditional diagnostics. In LTBI management, a major clinical hurdle is the inability to distinguish “stable latency” from “imminent progression” (Daniel et al., 2025). In QuantiFERON supernatants, a combination of malate and N-arachidonyl glycine exhibits excellent predictive potential (AUC 0.99), identifying individuals about to undergo reactivation before pathological signs emerge (Chen et al., 2021) (**Table 2**). This model allows for the identification of high-risk populations during the window when traditional microbiology remains negative.

**Table 2:** Evaluation of TB diagnostic and early-warning biomarkers based on lipid metabolic features.

Application scenario	Characteristic metabolites/lipid fingerprints	Diagnostic performance (AUC)	Core clinical significance	References
Latent reactivation risk prediction	Malate + N-arachidonyl glycine	0.99	QuantiFERON supernatant analysis; identifies disease risk before clinical onset	Daniel et al. (2025)
Early diagnosis of active TB	Phosphatidylcholine PC (15:0/17:1)	High Sensitivity	Levels correlate with disease severity; overcomes challenges in sputum collection	Sun et al. (2023)
Comorbidity risk identification	CE(20:0) + PC(14:0_20:4)	0.834	Accurately distinguishes between TB, CAD, and TB-CAD comorbidity	Zhao et al. (2025)
Dynamic evaluation of therapeutic efficacy	Lysophosphatidic acid LPA (0:0/16:0)	1.0 (100%)	Normalizes within weeks of treatment; superior to traditional sputum culture	Chen et al. (2021)

Lipid markers have also demonstrated unique diagnostic value in complex clinical settings (Chen et al., 2021; Fang et al., 2025; Zhu et al., 2025). For tuberculosis complicated by coronary artery disease (TB-CAD)—a form of comorbidity that is becoming increasingly common in an ageing society—a combination of specific lipid species, such as CE(20:0) and PC(14:0,20:4), enables accurate differential diagnosis with an AUC of 0.834 (Daniel et al., 2025). In terms of treatment efficacy monitoring, the plasma lysophosphatidic acid (LPA) (0:0/16:0) exhibits excellent kinetic responsiveness, with its concentration rapidly returning to normal within weeks of effective treatment; both its sensitivity and specificity are superior to traditional sputum culture conversion monitoring (Chen et al., 2021). By dynamically monitoring the evolution of these “lipid fingerprints”, clinicians can transition from standardised fixed-duration regimens to personalised precision therapy, which may shorten the treatment duration for some patients whilst also enabling the timely identification of individuals with poor treatment response and the adjustment of treatment regimens.

## Discussion

This review systematically examines the multi-level regulatory mechanisms of lipid metabolism during the dormancy and resuscitation of *Mtb*. From the molecular to the systemic level, lipid metabolism remains a central hub determining *Mtb*’s survival strategies and pathogenicity. At the bacterial level, *Mtb* responds to phosphate limitation through membrane lipid remodelling, defends against oxidative stress via the “Reductive Sink” mechanism, and achieves precise metabolic transitions from dormancy to resuscitation through the MtrA-OmamC regulatory axis; At the host-pathogen interaction level, *Mtb* secretes 1-TbAd to hijack lysosomal function, constructing a lipid-rich “metabolic niche”; at the systemic level, the PPE2 protein induces systemic metabolic dysregulation, creating favourable conditions for the reactivation of distant lesions. This cross-scale lipid metabolism regulatory network not only reveals the survival strategies enabling *Mtb* to persist long-term within the host, but also provides a unified metabolic framework for understanding the dynamic evolution of the tuberculosis disease spectrum. From the perspective of the continuous pathological spectrum defined by the ICE-TB consensus, the transition of lipid metabolic states may be the key molecular event driving the progression of the disease from a stable infection state to the initial state, subclinical state, and ultimately to the clinically manifest state.

Although significant progress has been made in recent years in the fields of lipid metabolism and tuberculosis reactivation, numerous scientific questions remain to be addressed. Firstly, existing studies are largely based on *in vitro* culture models or animal models, resulting in limited understanding of the actual metabolic dynamics within the human body. In particular, the ‘persistent’ bacterial population at different stages of the disease spectrum exhibits high heterogeneity, which manifests as variations in the microenvironment—such as oxygen tension, pH, and nutrient availability—alongside distinct metabolic sub-populations of Mtb within the same host, and the precise relationship between their metabolic characteristics and the tendency for reactivation remains unclear. Secondly, there is currently a lack of systematic research into how host factors influence *Mtb*’s lipid metabolic strategies and the risk of reactivation. Although the bidirectional causal relationship between TB and type 2 diabetes mellitus (T2DM) has been partially elucidated at the molecular level, the mechanisms of interaction between other metabolic diseases (such as obesity and non-alcoholic fatty liver disease) and tuberculosis remain to be explored in depth. Thirdly, technical bottlenecks persist in the transition from basic research to clinical translation. Although lipidomics testing shows great diagnostic potential, standardised, high-throughput, low-cost testing platforms have yet to be established; drug development targeting lipid metabolism is also in its early stages, and its efficacy and safety require large-scale clinical validation.

Achieving the WHO’s strategic goal of “Ending Tuberculosis” requires a fundamental understanding of and intervention in the survival strategies of *Mtb*. Future research should focus on four key areas: first, utilising single-cell multi-omics [such as single-cell RNA-sequencing of infected host cells (Lyu et al., 2024)] and spatial metabolomics techniques to elucidate the metabolic heterogeneity of dormant bacterial populations and identify specific markers for “high-risk” subpopulations poised to resuscitate; Second, developing new host-directed therapy (HDT) strategies, such as TRPML1 agonists to restore lysosomal function and metabolic modulators to enhance immune clearance, in combination with traditional antibiotics to shorten treatment duration; Third, developing specific inhibitors targeting key metabolic nodes such as ICL, lipases, and WhiB3/MtrA to fill the therapeutic gap in the treatment of dormant infections; Fourth, establishing a standardised lipidomics testing platform; validate the predictive efficacy of metabolic biomarkers through multicentre prospective studies; and integrate multi-omics data to construct personalised risk prediction models. As a core driving force throughout the entire process of latent infection, reactivation and transmission, lipid metabolism provides a new strategic entry point for tuberculosis prevention and control. By precisely disrupting the essential lipid pathways required for bacterial survival, reversing its systemic hijacking of the host’s metabolic homeostasis, and combining this with early warning and precision treatment based on metabolic signatures, this comprehensive prevention and control strategy—in synergy with innovative approaches such as rapid diagnosis and new drug research—will lay a solid foundation for ultimately achieving the goal of “ending tuberculosis”.
